# Appraisal for the Potential of Viral and Nonviral Vectors in Gene Therapy: A Review

**DOI:** 10.3390/genes13081370

**Published:** 2022-07-30

**Authors:** Muhammad Hammad Butt, Muhammad Zaman, Abrar Ahmad, Rahima Khan, Tauqeer Hussain Mallhi, Mohammad Mehedi Hasan, Yusra Habib Khan, Sara Hafeez, Ehab El Sayed Massoud, Md. Habibur Rahman, Simona Cavalu

**Affiliations:** 1 Department of Pharmaceutics, Faculty of Pharmacy, University of Central Punjab, Lahore 54000, Pakistan; hmdbut@ucp.edu.pk (M.H.B.); abrar.ahmad@ucp.edu.pk (A.A.); rahima.khan888@gmail.com (R.K.); 2 Department of Clinical Pharmacy, College of Pharmacy, Jouf University, Sakaka 72341, Saudi Arabia or thhussain@ju.edu.sa (T.H.M.); or yhkhan@ju.edu.sa (Y.H.K.); 3 Department of Biochemistry and Molecular Biology, Faculty of Life Science, Mawlana Bhashani Science and Technology University, Tangail 1902, Bangladesh; mehedi.bmb.mbstu@gmail.com; 4 Department of Biotechnology, Quaid-i-Azam University, Islamabad 45320, Pakistan; sarahafeez17@gmail.com; 5 Biology Department, Faculty of Science and Arts in Dahran Aljnoub, King Khalid University, Abha 62529, Saudi Arabia; ehabma@kku.edu.sa; 6 Research Center for Advanced Materials Science (RCAMS), King Khalid University, Abha 61413, Saudi Arabia; 7 Agriculture Research Centre, Soil, Water and Environment Research Institute, Giza 3725004, Egypt; 8 Department of Global Medical Science, Wonju College of Medicine, Yonsei University, Wonju 26426, Korea; pharmacisthabib@gmail.com; 9 Faculty of Medicine and Pharmacy, University of Oradea, Pta 1 Decembrie 10, 410087 Oradea, Romania

**Keywords:** gene delivery, viral vectors, nonviral vectors, gene expression, transgene, gene therapy

## Abstract

Over the past few decades, gene therapy has gained immense importance in medical research as a promising treatment strategy for diseases such as cancer, AIDS, Alzheimer’s disease, and many genetic disorders. When a gene needs to be delivered to a target cell inside the human body, it has to pass a large number of barriers through the extracellular and intracellular environment. This is why the delivery of naked genes and nucleic acids is highly unfavorable, and gene delivery requires suitable vectors that can carry the gene cargo to the target site and protect it from biological degradation. To date, medical research has come up with two types of gene delivery vectors, which are viral and nonviral vectors. The ability of viruses to protect transgenes from biological degradation and their capability to efficiently cross cellular barriers have allowed gene therapy research to develop new approaches utilizing viruses and their different genomes as vectors for gene delivery. Although viral vectors are very efficient, science has also come up with numerous nonviral systems based on cationic lipids, cationic polymers, and inorganic particles that provide sustainable gene expression without triggering unwanted inflammatory and immune reactions, and that are considered nontoxic. In this review, we discuss in detail the latest data available on all viral and nonviral vectors used in gene delivery. The mechanisms of viral and nonviral vector-based gene delivery are presented, and the advantages and disadvantages of all types of vectors are also given.

## 1. Introduction

Medical research has identified approximately 3400 genes that are somehow associated with diseases at the moment [1]. Most of these diseases are life-threatening or wearying and do not have any potential treatment options available. With the advancement in genome editing, this number is expected to increase manifold. This medical concern can be combatted by gene therapy which can target these genes responsible for causing diseases in humans. It is an adept treatment strategy that is intended to treat the disease itself and not just the symptoms by silencing problematic genes, inserting new genes that bring about the desired gene expression, or relacing ambiguous and problematic genes with healthy genes. However, the implementation of these strenuous strategies comes with many challenges, including the deficient efficiency and safety of these innovative approaches and the difficulty in operations of gene handling and delivery.

Genetic material in the form of nucleic acids of different types of DNA and RNA is very vulnerable in naked form. Naked genetic material can be degraded by the action of different materials found in biological fluids and also initiate unwanted immune responses, which can result in their deactivation and elimination, leading to the failure of effective therapeutic action. This has led to the need for suitable vectors that can protect genes from degradative action of the biological environment, are capable of crossing biological membranes, and allow better intracellular targeting. Although many gene therapy products have arrived on the pharmaceutical market, there is still more research needed in the field of vectors for gene delivery in order to take the efficiency of gene therapy to a new level of therapeutic effectiveness [2].

The biggest hurdle faced by medical research in gene therapy is the availability of effective gene-carrying vectors that meet all of the following criteria:

- Protection of transgene or genetic cargo from degradative action of systemic and endonucleases,
- Delivery of genetic material to the target site, i.e., either cell cytoplasm or nucleus,
- Low potential of triggering unwanted immune responses or genotoxicity,
- Economical and feasible availability for patients [3].

**Figure 1:**
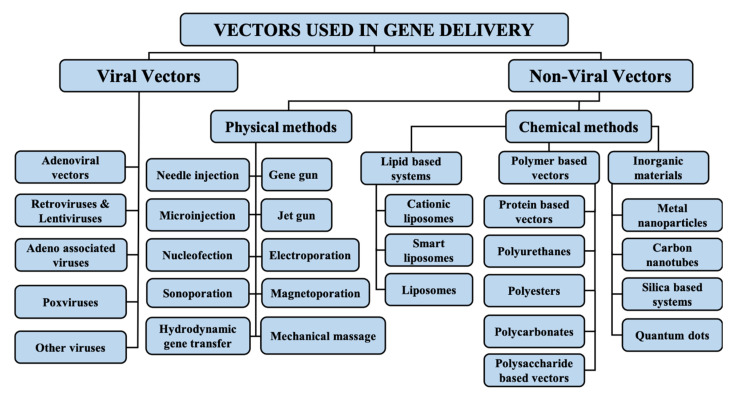
Types of vectors used in gene delivery.

Vectors used in gene therapy are generally divided into two main categories, i.e., viral and nonviral vectors, as further explained below. An overview of the types of vectors used in gene delivery is given in Figure 1.

The choice of the vector to be used in gene delivery depends upon the type of genetic material to be delivered, the type of gene therapy strategy being pursued, the amount of the genetic material to be delivered, and the route of administration. Both viral and nonviral vectors have their own unique advantages and disadvantages. Viruses provide good transfection efficiency and sustainable gene expression, and they protect the gene from degradation; however, they are vulnerable to immunogenicity, can be highly toxic, have poor targeting potential, and have very high costs. On the contrary, nonviral vectors are relatively less toxic, capable of transferring large quantities of genetic material, and easy to prepare, while they do not trigger unwanted immune reactions. However, they also pose some disadvantages such as high vulnerability to extracellular and intracellular barriers, decreased transfection ability, and much lower expression of the transgene. Thus, both types of vectors have their own benefits and drawbacks. However, they allow chemical and physical modifications by attachment of targeting ligands and other promoters in the form of proteins and peptides, which aid them in the process of gene delivery [4].

A large number of genes have been identified that are involved in causing numerous diseases of acquired and inherited nature, and, with advancements in technology, new and innovative vectors and transfer techniques are emerging, which greatly enhance the potential of gene therapy in treating such disorders. The success of a particular gene therapy process highly depends upon the vector and gene transfer technique used.

In this review, we discuss the types of viral and nonviral vectors used in gene delivery in detail, along with their advantages and disadvantages, and the mechanisms via which viral and nonviral systems bring about gene delivery to target cells.

## 2. Viral Vectors for Gene Delivery

Over the course of millions of years, viruses have evolved and adapted to changes in the biological environment which has allowed them to survive and replicate in host cells. Using this feature of viruses, gene therapy research has developed new approaches utilizing viruses and their different genomes as carriers and vectors for the delivery of genes, nucleic acids, and other genetic material to cell target sites. The major advantage that the use of viral vectors has brought to gene therapy is their ability to protect transgenes from biological degradation and the capability to efficiently cross cellular barriers. The first clinical trial conducted on gene therapy in 1999 utilized viral vectors for the treatment of severe combined immunodeficiency disorder and proved to be successful. According to *The Journal of Gene Medicine*, up until November 2017, more than 68% of the clinical trials conducted on gene therapy utilized viruses as vectors for gene delivery [5]. The promising use of viral vectors in gene therapy has recently been backed by the approval of several gene products based on viral vectors by the US FDA. Many more products using viruses as delivery vehicles are in the phases of clinical trials or process of approval.

Although the broad application of viral vectors in gene therapy has led to the belief that they are safe for human use despite their infection potential, their harmful nature is still of a major safety concern, which is important to address while developing gene therapies. For this purpose, scientists have come up with some engineering strategies that are aimed at enhancing the safe use of viral vectors without limiting their efficiency. These include avoidance of viral replication, promotion of viral inactivation, and attenuation of natural toxicity of viruses [6].

Another feature of viruses that makes them suitable for gene delivery is their occurrence in a wide range of types and species that have varying properties of size, morphology, type of genetic material, and natural tropism, allowing more variety to choose from as per the requirements of the specific gene therapy. There are several criteria on the basis of which viruses can be classified. These include the presence of envelope, symmetry of viral capsid, nature of viral genetic material, i.e., DNA or RNA, replication site of the virus, i.e., nucleus or cytoplasm, and virion size [7].

The choice of a specific viral vector for gene therapy is made by keeping in view the above criteria of viral classification and the advantages and disadvantages of viral vectors in order to ensure efficient gene delivery. Some advantages and disadvantages of viral vectors in gene delivery are mentioned in Table 1 [8].

**Figure 2:**
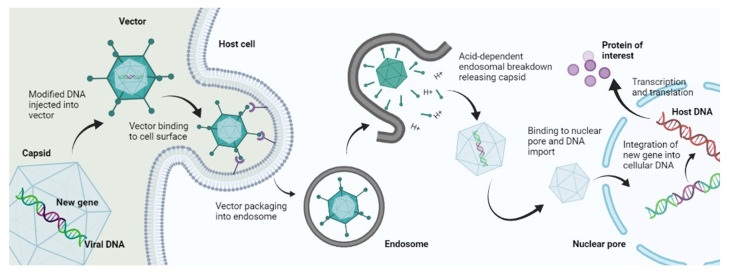
Mechanism of viral gene delivery.

The mechanism of viral gene delivery (Figure 2) starts with the incorporation of the transgene into the viral DNA, and then this modified DNA is injected into the viral vector. This vector, upon reaching the target site, attaches to the receptors found on the cell membrane of the target cells. After cellular internalization, the vector is packed into endosomes, followed by an acid breakdown of these endosomes that release the capsid containing the modified DNA. This capsid then travels to the nucleus and binds to nuclear pores to enter the nucleus, where the modified gene is integrated into the DNA of the target cell. After that, transcription and translation occur, which form the protein of interest and bring about gene expression [9]. This mechanism is followed by Lentiviruses and most Retroviruses [10]. However, some viral vectors do not bring about gene delivery by integration into host genome such as Adenoviruses; they simply deliver the genetic material into the cytoplasm or nucleus, and transgene expression occurs from there [11].

The different types of viral vectors used in gene therapy are further explained below.

### 2.1. Adenoviral Vectors

Adenoviruses are among viruses that were studied first and foremost for the purpose of gene therapy. They were proposed to be used as gene delivery vectors about 20 years ago [12]. They contain a DNA genome that is double-stranded and has a size of 35 kb. They are nonenveloped viruses. Attenuation of adenoviruses is achieved by deletion of fragments of their genome that specifically code for early proteins. There are three generations of adenoviral vectors that are based on the level of attenuation achieved by the deletion of genes. In the first-generation adenoviral vectors, the E1A and E1B genes are deleted. In the second-generation adenoviral vectors, a large number of the early genes are deleted. In third-generation adenoviral vectors, the complete genome or genetic information of the virus is deleted, which is why they are also called gutless vectors [13].

The adenoviral genome is quite large in size, and the prospect of complete genome deletion greatly renders these viruses a high coding capacity. First-generation vectors can allow ~3.2 kb of genome insertion, while third-generation vectors allow up to 30 kb. An advantage of adenoviral vectors is that there is a very negligible possibility of integration of their genome into the genome of the host cell, which makes them rather safe and nontoxic for use. However, long or sustainable gene expression is difficult to achieve with adenoviruses because their life cycle is not adapted to it [14].

Originally, scientists believed that adenoviruses could serve as vectors for a large variety of therapies ranging from gene delivery to hereditary disorders and regenerative therapy. However, it turned out that several toxic properties of adenoviruses render them unsuitable for this purpose. These include their ability to trigger severe immune responses due to their highly immunogenic capsids. They are also more vulnerable to attachment with blood-circulating proteins, ligands, and other blood cells, which can cause viral inactivation and hinder the system delivery of transgenes [15]. Moreover, if these viruses are administered via a systemic route in large doses, then they can result in a severe inflammatory response which can be life-threatening. This toxic potential of these viruses has limited their use as vectors in gene therapy where local administration of transgene is required, for example, against malignant tumors [16].

Despite these disadvantages of adenoviral vectors, researchers have devised new ways of modifying adenoviral vector systems to improve their gene transfection ability. These include extensive global genome modification with the deletion of almost all genes except those required for replication and packaging. This reduces the toxicity of adenoviral vectors, rendering them with a very high cloning capacity, i.e., ~36 kb, and the ability to deliver multiple transgenes or genomic loci at a time. The formed adenoviral vectors are called helper-dependent or high-capacity adenoviral vectors (HAdVs). Several studies reported a very prolonged transgene expression and high reduced immunogenicity of adenoviral vectors by employing this technique [17,18,19]. However there are several challenges associated with the use of HAdVs for gene transfer which include increased immunogenicity, transient expression of transgene, triggering of potent immune and inflammatory reactions, and pre-existing immunity in cancer patients [20,21]. Researchers have, however, developed some ways of overcoming these challenges which include altering the tropism of HAdVs, preparation of vector chimeras, and combination immunotherapy treatments [22,23,24,25].

### 2.2. Retroviruses and Lentiviruses

These are RNA viruses, the replication of which is based on reverse transcription of RNA to DNA followed by its integration into the host genome. Earlier in the 1990s, retroviruses were studied for their potential to be used in gene therapy for the treatment of diseases caused by defects in a single gene and not a segment of a genome. An example of this is the use of these viruses in gene therapy for the treatment of severe combined immunodeficiency caused by a problem in the gene that codes for the enzyme adenosine deaminase [26]. For this purpose, the γ-retrovirus murine leukemia (MLV) virus was used as a vector. The specificity of retroviruses to integrate at a specific portion of the host genome resulted in their choice as vectors being a failure. This is because MLV had a different genome insertion focal point than the target, which led to oncogene expression leading to genotoxicity and the development of leucosis in five out of 20 patients who participated in the clinical trial. To overcome this problem, another group of retroviruses called lentiviruses was explored for their potential to be used as vectors in gene therapy. The genomic insertion point of these viruses was different from MLV vectors; thus, they showed a decreased occurrence of genotoxicity [27,28].

Retroviruses have various benefits over different vectors. The main benefit that retroviral vectors offer is their capacity to change their ssRNA genome into a dsDNA particle that steadily incorporates into the genome of its host cells. This element empowers the retroviral vectors to alter the nuclear genome of host cells and bring about gene expression [29,30]. At present, retroviral vector-mediated gene therapy has been revived with the improvement of a new retroviral vector class called Lentiviruses (LV). The LV have the special capacity among RV to taint noncycling cells. Vectors obtained from LV have given a significant jump in gene editing and gene transfer, and they present new roads to accomplish huge degrees of gene delivery in vivo. Lentiviruses include the human immunodeficiency virus HIV. Although lentiviruses show a much lower extent of mutagenesis than MLV retrovirus vectors, their safety level is still of great concern for use as vectors in gene delivery [31,32]

Control levels for the utilization of retroviruses are resolved in light of the cell types they infect. BSL-1 is fitting for RV that do not contaminate human cells. BSL-2 is important if they are utilized to contaminate human cells [33]. The essential danger with the utilization of RV emerges from their capacity to coordinate into the host cell chromosome, which raises the chance of insertional mutagenesis and oncogene initiation [34]. Formation of RV capable of replicating in target cells or tissues is the essential disadvantage connected with the utilization of retroviral vectors. Appraisal of this chance is basic in deciding the security related with the utilization of retroviral vector frameworks. Furthermore, the scope of the target cell accession of the vector is also a security issue [35].

Use of a viral envelope that can infect cells from numerous species increases both the risk of forming RV capable of replicating and the likely risk of any subsequent infection, which could spread from one animal varieties to another. Future examinations that use retroviral vectors in quality treatment tests should investigate more secure methodologies that center around the dangers related with in vivo recombination, age of mosaic RV, and storage of viral genetic data for longer periods of time [36].

The utilization of LVVs in research is as yet connected with potential risks, and the drawn-out security of these clinical mediations is as yet being assessed. While lentiviral frameworks are obtained from HIV, their association across various plasmids and the erasure of numerous HIV proteins brings down the probability of creating a replication-competent virus. These vector frameworks are dealt with at BSL-2 [37]. The impediments of involving LVVs in preclinical trials today are for the most part because of deficient strategies for the creation of high-titer infection stocks and the security concerns connected with their HIV origin, notwithstanding the designing of packaging cell lines and erasures of viral replication genes. One way to deal with these security issues has been to foster LV vectors unequipped for replication in human cells. Despite the fact that LVVs are less connected with insertional mutagenesis than other RV, these vectors actually give proof of off-target effects [38].

### 2.3. Adeno-Associated Viruses

Belonging to the viral family of Parvoviridae, adeno-associated viruses (AAV) are DNA viruses having a single strand, which are small and nonenveloped. These viruses are nonautonomous, which makes them incapable of replicating when adenovirus is not present. Naturally, these viruses do not integrate into host cell genomes and remain inactive after infecting humans. AAV genomes integrate into the host’s genome in 0.1% of the cases via insertion into a specific portion of chromosome 19. Vectors based on these viruses have not yet been shown to cause genotoxicity because of the lack of insertion of viral genome into the host cell genome. However, this property has resulted in a side-effect against use as gene delivery vectors, i.e., the level of transgene expression is reduced in dividing cells where the AAV genome is decreased. However, this property has allowed them to be used for gene therapy where target cells are slow-dividing, such as cardiomyocytes [39].

These viruses also have a less immunogenic and toxic capsid as compared to other types of viral vectors such as adenoviruses or poxviruses. AAV shows a negligible immune response upon systemic administration and is stable in blood to a great extent. The low level of AAV vector side-effects and decreased toxicity potential have led to them becoming the safest viral vectors for gene therapy that can provide a good transgene expression [40].

### 2.4. Poxviruses

Belonging to the Poxviridae family of viruses, they are the most complicated and largest of all viruses that infect humans and cause diseases. Their genome is double-stranded and has a size of approximately 180–220 kb. The smallpox virus is the most popular virus belonging to this family and represents its group very well. The vaccinia virus belonging to the poxvirus family is the virus which was used for the development of smallpox vaccine. There are two features unique to this virus: its capability to carry out its life cycle in the cell cytoplasm due to which it does not insert its genome into the host cell genome, and its occurrence in two different infectious variants i.e., an intracellular mature virus (IMV) and an extracellular enveloped virus (EEV) [41].

The efficient life cycle of the vaccinia virus allows its use in gene therapy for cancer. Moreover, the virus also has a reasonable cloning capacity. Its capacity is up to 25 kb if no part of the viral genome is removed, and this capacity can increase up to 75 kb if some parts of the viral genome are deleted. However, the complexity of the viral structure and genome makes it difficult to be used in gene therapy. Nevertheless, there are several ongoing clinical trials of recombinant vaccinia viral vectors for oncolytic gene therapy [42].

### 2.5. Other Virus

Most human, animal, and bird viruses can now be subjected to genetic engineering and modifications of the viral genome, collectively called reverse genetics. This property makes them feasible for genome changes and engineering for transgene delivery in gene therapy. Some herpes viruses are being used in treatment of gene-related CNS disorders due their property of being neurotropic [43]. Moreover, some baculoviruses that belong to the Baculoviridae family are also being explored for their potential to be used as vectors in gene delivery. These viruses possess a reasonable cloning capacity of about 38 kb, and they allow insertion of about 100 kb of genomic material in their capsid. These viruses also do not replicate in mammalian cells, which reduces their risk of causing toxicity. Several studies are exploring the use of these viruses for gene delivery in treatment of certain lymphomas [44].

Several vector-shielding strategies have been devised by researchers to protect the viral vectors from interacting with blood components and causing unwanted immunogenic reactions. One of them includes chemical capsid modification with compounds, e.g., thiol-directed genetic capsid modification. Others include attachment of adapter molecules for targeting purposes, introduction of cysteine moieties or peptides in hexon or fiber of viral capsid, introduction of point mutations, or fiber pseudotyping with whole fiber or knob of different serotype. These techniques have proven to be successful in reducing liver tropism and immunogenicity potential of viral vectors, most importantly adenoviral vectors [45].

## 3. Nonviral Vectors for Gene Delivery

Although viral vectors provide a sustainable gene expression, their developmental process is significantly tedious, which makes them difficult to use. Furthermore, the toxic properties of viral vectors such as triggering an immune response and the potential of insertional mutagenesis have raised many safety concerns on their use and impeded their development. For this purpose, researchers have been looking to find suitable vectors based on nonviral systems that can provide sustainable gene expression without triggering unwanted inflammatory and immune reactions, and that are considerably nontoxic. Nonviral vectors are very cost-effective, versatile, non-immunogenic, and stable, and they have a high loading capacity which has led to them gaining significant importance in gene delivery research [46].

**Figure 3:**
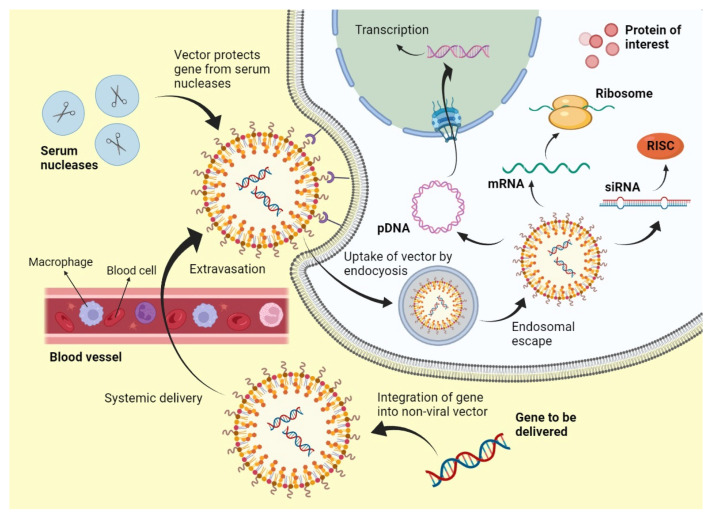
Mechanism of nonviral gene delivery.

The types of nonviral vectors in gene delivery are generally divided into two main groups, i.e., physical and chemical systems. Physical methods include procedures such as electroporation, sonoporation, magnetoporation, microinjection, needle injection, and gene gun, whereas chemical methods consist of polymer and lipid-based systems and several inorganic materials. Most of these chemical systems are cationic. They are capable of combining with negatively charged DNA by the use of electrostatic interactions. This classic interaction leads to an overall positive charge on the vector–gene complex. This positively charged entity then binds to the negatively charged molecules of the cellular membrane and crosses it, leading to cell internalization. After this, they have to escape degradation by endosomes and lysosomes and deliver the transgene to the target site, i.e., cell nucleus (in the case of DNA) or cell cytoplasm (in the case of mRNA and siRNA) [47]. This mechanism of nonviral gene delivery is presented in Figure 3 [48].

### 3.1. Physical Methods for Nonviral Gene Delivery

**Figure 4:**
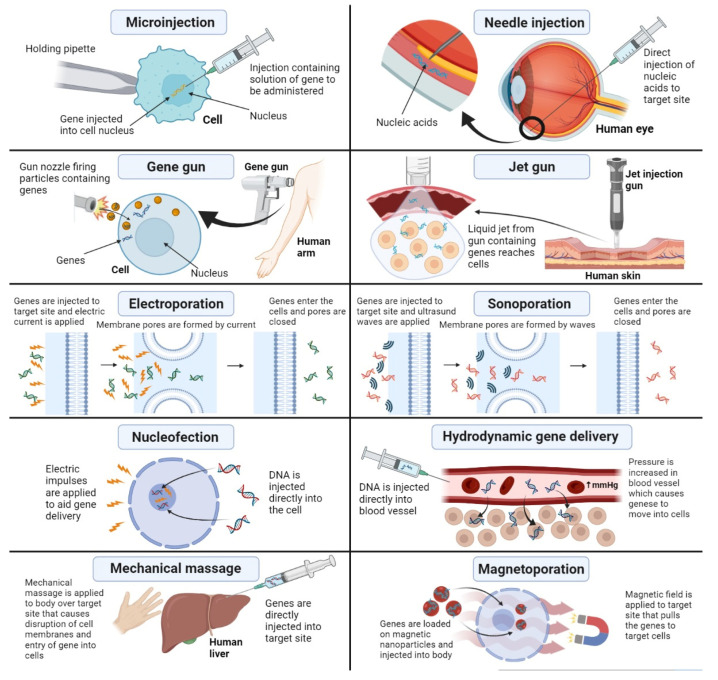
Physical methods of nonviral gene delivery.

The problems of gene delivery associated with viral vectors have received great attention from researchers. A large number of clinical trials have been conducted on finding new and innovative physical approaches to gene delivery that are better than other viral vectors and chemical approaches. These physical methods are of immense importance as they are capable of bypassing the extracellular and intracellular barriers associated with gene delivery, thus providing greater gene transfection ability, which is compromised in approaches to gene delivery due to these barriers. Most common barriers to gene delivery include the interaction of vector with blood components, degradation by serum nucleases, cellular uptake, and endosomal escape. Physical methods of gene delivery are capable of encompassing all these barriers as they involve physical techniques that deliver the vector and gene to the target site without having to cross the extracellular and intracellular environments. In this article, we describe all the latest and innovative physical methods of gene delivery which include microinjection, needle injection, gene gun, jet gun, electroporation, sonoporation, nucleofection, hydrodynamic gene delivery, mechanical massage, and magnetoporation; the advantages and disadvantages of all these methods are given in Table 2, and they are presented diagrammatically in Figure 4 [49,50].

#### 3.1.1. Microinjection

Microinjection is a process whereby the transgene is directly injected into the inner cell target. This method was first demonstrated and described by the medical researcher Barber. In this technique, a very small-sized needle, i.e., 0.5–5 µm, is filled with a solution containing the cargo genetic material. This solution is then injected directly into a single cell by continuous observation under a microscope [51]. The microinjection technique is quite simple and economical and has a great advantage of delivering large-sized DNA or genetic material. It is a nontoxic, biocompatible, and reproducible way of effective gene delivery. This technique does come with several downsides, which include strict handling techniques, the requirement of individual cell manipulation, and a low level of gene expression and gene persistence. Another disadvantage of this technique is that a small volume of genetic material can be injected in order to avoid disorganization of cellular membrane and intracellular environment. This method is also limited to cells that are relatively larger in size such as oncocytes [52].

#### 3.1.2. Needle Injection

This involves the direct injection of naked DNA or gene material to target site. The needle injection technique was first reported by Wolf and coworkers in 1990, who administered an intramuscular injection of naked DNA directly into myofibers of mouse muscle [53]. The remarkable simplicity of this procedure renders it feasible for use in gene therapy where the gene is targeted to a single region, such as eye cells, brain cells, or blood. The conducive nature of this technique allows it to be used in DNA-based gene therapy and vaccination procedures. It can allow delivery of not only naked DNA but also different types of RNA such as siRNA [54]. The method is also much less toxic and much friendlier to the biological environment. However, there are some drawbacks associated with the needle injection technique, which include a very low gene expression in the case on IV injected of naked genetic material. This problem has now been resolved by the development of a high-pressure gene delivery procedure called hydrodynamic gene transfer, which is later explained in this section [55].

#### 3.1.3. Jet Gun

The jet injection method of drug delivery has been in practice in medicine since 1947. It is a needle-free method that is capable of inserting and dispersing the therapeutic material or gene in target cells [56]. This device is capable of creating a very fine jet of genetic material that is delivered to the target site at a very high pressure. The amount of pressure exerted determines how evenly the genetic material is distributed. The jet injection can cause some tissue damage, but it can be tolerable as long as the desired gene expression and gene delivery are achieved. An example of the process conditions applied for gene therapy in cardiac disorders is as follows: 110 m/s velocity of nozzle jet, 20–25 cm of distance, 100–500 µL volume of injection, and 150–250 kPa of pressure. The selection of the jet parameters greatly influences the depth to which tissue penetration is achieved and how efficient this technique proves to be. The flexibility of this process in a way that it allows adjustment of these parameters greatly helps in reducing tissue damage as a result of this technique [57].

The jet gun or jet injection technique has been employed in gene therapy applications such as genetic vaccines and targeting of suicide genes that encode DNA for the purpose of antitumor therapy. Many skin diseases have also been corrected genetically by utilizing the jet gun method. The jet gun process has proven to be useful in targeting many different types of tissues such as the skin, mammary tissues, muscles, and fat tissues. This method has yet not shown any potential adverse effects except a few, which include minor bleeding at site of injection, local inflammation or hyperthermia, and edema [58].

#### 3.1.4. Gene Gun

This method, also known as ballistic gene delivery or ballistic DNA injection, is based on the use of DNA or genetic material-coated particles that are bombarded on tissues or target cells in gene therapy. This process is very widely used to transfer genetic material to different target cells such as the mucosa, skin, tumor cells, or surgically exposed body tissues. It employs the use of heavy metals such as tungsten and gold as particles which are first coated with the genetic material to be delivered and then pressurized so they can effectively cross the cell membranes and achieve cellular internalization, where they can deliver the genes or nucleic acids to the cell cytoplasm or nucleus. There are different physical properties of these payload particles that can impact the efficient transfer of genes to target sites. These include the particle size, surface morphology, shape, dose, and speed of release [59].

This method, albeit safe and convenient, does have several disadvantages which pose a challenge to its use in gene transfer. The biggest drawback of this system is that it provides very few milliliters of depth of gene transfer, as a result of which it is more suitable for therapies where localized injection is required. Furthermore, the gene expression achieved by this method is transient, which has led to its use being limited to cells that actively divide and have a fast proliferation rate. Researchers have developed several ways of improving the efficiency of this technique, which include efficient carrier preparation methods, reduction in damage to tissues, and reduction in time consumption by the process [60].

#### 3.1.5. Electroporation

First used in 1982 [61] and 1991 [62], electroporation, also known as electrofection or electropermeabilization, is a process that has been widely used to deliver genes to target sites for both in vivo and in vitro purposes. This process is mostly employed for transfer of materials that are impermeable to cell membranes.

This technique works by imposing an electric current on the cells at the target site, which results in the formation of temporary pores of nanometric size. This allows the negatively charged DNA or other genetic materials which are otherwise nonpermeable through membranes to cross through these pores and deliver the genetic material to cell cytoplasm or nucleus, thus bringing about effective gene delivery. The duration and strength of electric pulses applied to target cells vary greatly on the type of cells being targeted. An example of this is the skeletal muscles, where a relatively high electric current is first applied that results in formation of membrane pores. After this, some low-intensity pulses are applied, which guide the genetic material across these pores through an electrophoretic effect [63].

A large variety of electrodes are available to be used and selected depending upon the target organ. These include spoon electrodes, multielectrode arrays, customized defibrillator pads, caliper electrodes, plate electrodes, and nonpenetrating charged needles [64].

The formation of pores by electric pulses occurs for a very short period of time, which makes it necessary for genetic material to be present at that time in order to ensure its effective passage across these pores to be able to reach cytosol. This process is largely employed for in vitro nucleic acid delivery, as well in some in vivo applications for the treatment of tumors of skin and liver. However, it is largely limited to local targeting and also has a great potential of cell damage and cellular toxicity [65].

#### 3.1.6. Nucleofection

Similar to the method of electroporation, nucleofection is a rather more advanced and fruitful technique as compared to electrofection. In this technique, electric pulses are applied to target cells, which result in the formation of membrane pores, through which genetic material can pass and reach the cell cytosol, leading to gene delivery. The difference lies in the fact that, in nucleofection, the exogenous material is directly injected inside the cell, which increases the efficiency of the process and allows gene transfer to cells, which are comparatively hard to transfect.

The main advantages of nucleofection are that it is not very time-consuming, and it requires a very small number of cells for delivery. However, some studies have reported a high mortality rate in clinical trial participants of this type of gene therapy. Therefore, further research is required in this respect to explore more prospects of this technique while focusing on reduction of its toxic potential [66].

#### 3.1.7. Sonoporation

Sonoporation, also known as sonofection, works using the same principle as electroporation. It uses ultrasound waves to create pores in the cell membrane from which genetic materials can cross into the cell and bring about gene expression. Usually, high-intensity ultrasound waves are used for this process. However, the effectiveness of the process and the size of genetic material that can cross the pores greatly depend on the intensity of ultrasound waves applied. The main action of ultrasound waves is brought about by the process of cavitation. In this process, an acoustic field mediates the formation and collapse of bubble-like voids that have very low pressure. These bubbles or voids bring about an oscillating motion and grow, as a result of which they implode and release large amounts of energy. This leads to sonoporation and a rise in the surrounding temperature and pressure. After this, pore formation leads to the transfer of genetic material across cell membranes leading to internalization of gene cargo to cytosol [67].

Researchers have been trying to bring about an improvement in the cavitation process by the use of nucleation agents in the form of ultrasound contrast and agents and microbubbles, which cause enhanced membrane permeability and, thus, achieve efficient transfer of genes. The main advantage of this process is that it is noninvasive and, thus, can be used in cases where gene therapy is nonlocalized or targeted to deeper locations [68]. This technique can also be used for local gene therapy. In such cases, the contrast agent and genetic material are transferred to the bloodstream, after which ultrasound waves are applied. The contrast agent is incorporated with specific binding agents or ligands that target the specific tissue for which that ligand has affinity, thus allowing local gene delivery [69].

#### 3.1.8. Hydrodynamic Gene Transfer

In this technique, hydrostatic pressure at a very high rate is utilized as a driving and mediating force that allows transfer of genetic materials across cell membranes. Sufficient expression of the transgene in organs such as the liver, lungs, heart, and kidneys has been observed upon injection of a volume of DNA solution that is more than 8% of the body weight at a very fast rate in the mouse tail vein. The gene expression was found to be highest in the liver. The injection of the DNA solution at a very fast rate results in a buildup of pressure at the site of injection, which causes temporary pore formation in cell membranes, allowing the DNA material to cross through these pores and bring about gene expression intracellularly [70].

Although this process has proven to be beneficial for gene delivery in rodents, it has still not been used broadly in humans for gene transfer. Furthermore, the high-pressure buildup in hepatocytes can cause an increase in the functioning of hepatic enzymes, which can indirectly lead to cardiac dysfunction and congestion of the liver when very large volumes of genetic solution are injected. Researchers have come up with the use of catheters for transfer of genes in larger animals such as pigs to overcome the drawbacks of this technique. Although they have observed improved results, there is still much exploration required to make this process feasible for use in gene delivery to humans [71].

#### 3.1.9. Magnetoporation

This process, also known as magnetofection or magnetic transfection, makes use of a magnetic field to guide movement of genetic material across cell membranes into the cytosol. This magnetic field acts on vectors based on nucleic acids that are combined with magnetic nanoparticles. These magnetic vectors are prepared by combining lipid- or polymer-based gene vectors with nanoparticles that are magnetic in nature. Usually, iron oxide is used to bring about the magnetic property in nanoparticles. An external magnetic field is applied to drive the gene-carrying magnetic vector toward the specific target cells where gene expression is required [72].

Studies have shown that the application of a magnetic field enhances the property of genetic material to cross the cell membrane [73], and it can also enhance the degree of contact of genes in the cells internal environment, which brings about enhanced transfection and gene expression [74].

Magnetoporation has several advantages over electroporation. The most important is that this process does not require the direct contact of electrodes with cells of the target site, which makes it more convenient and noninvasive. Furthermore, this process is much less expensive and time-consuming, and it has a greater potential to penetrate deeper tissues and anatomical regions that are otherwise inaccessible with electroporation. Research has shown the application of magnetoporation in gene delivery to cardiac tissues [75], to cells of tumors and melanomas [76], and in pulmonary metastasis [77].

#### 3.1.10. Mechanical Massage

Liu and coworkers reported an enhanced gene expression by applying a mechanical massage at the site of injection after injecting naked DNA into the liver cells of mice. They explained the phenomenon in this way that application of light pressure through the massage causes temporary disruption of liver cell membranes, which provides opportunity for the genetic material to cross the membrane and enter the cell cytosol to provide gene expression [78]. Using this procedure for administration of hepatic growth factor for the treatment of fulminant hepatic failure induced by endotoxins resulted in a much-improved therapeutic effect. However, this process has only been tested on small animals such as mice and not humans. Further research is required to make this process feasible for humans [79].

### 3.2. Chemical Systems for Nonviral Gene Delivery

Recently, chemical nonviral vectors have gained immense popularity in the field of gene delivery. These systems have numerous advantages over viral systems such as immune privilege, safety, ability to transfect large quantities of genetic material, reduced toxicity, and simple preparation. Most of these systems are cationic in nature and comprise different types of cationic polymers, cationic lipids, and other inorganic materials including carbon nanotubes, quantum dots, metal nanoparticles, and silica-based systems. These cationic molecules are capable of forming strong complexes with negatively charged nucleic acids of genes and other genetic material by using electrostatic forces. These complexes, when combined with other targeting ligands and promoters, are capable of overcoming gene delivery barriers such as cellular uptake, endosomal escape, and delivery of gene to cytoplasm or nucleus. They also efficiently protect the genetic material from environmental degradation and have great potential to achieve an unhindered gene expression that is persistent and sustainable [80]. The types of chemical systems of nonviral gene delivery, including various polymers, lipids, and inorganic materials, are explained in detail below.

#### 3.2.1. Polymer-Based Nanovectors

**Figure 5:**
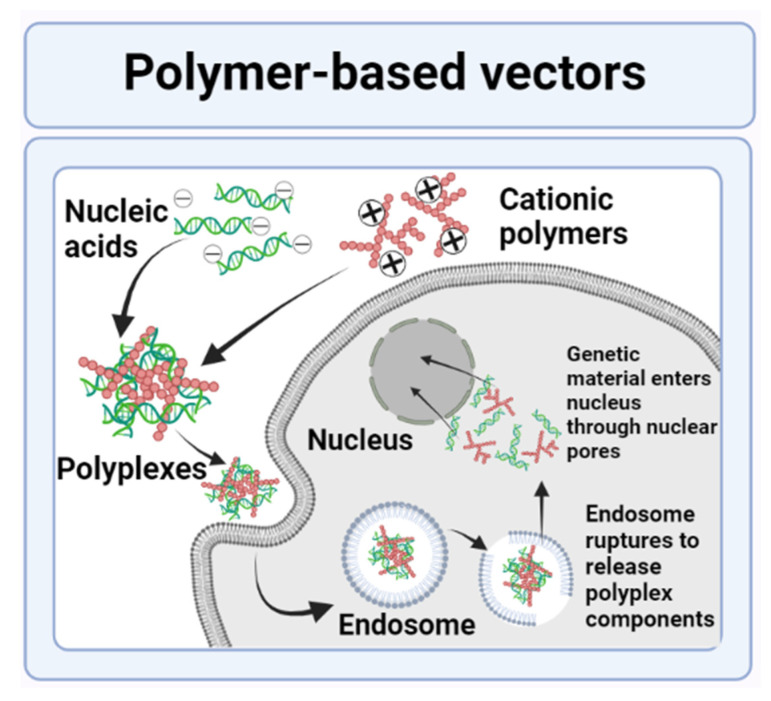
Gene delivery by polymer-based nonviral vectors.

Polymers are complex and large compounds made by bonding together of a large number of monomers or repeated units. These polymers are generally classified into two main categories, i.e., natural or synthetic polymers. Examples of natural polymers include some proteins, chitosan, and peptides. Examples of synthetic polymers include cyclodextrins and polyethyleneimine. The majority of these polymeric systems used in gene delivery are cationic in nature. They are capable of combining with negatively charged DNA or genetic material via the use of electrostatic interactions. These leads to an overall positive charge on the vector gene complex, also called a “polyplex”, which can easily cross the cell membranes and deliver genetic material to the target cytoplasm or nucleus [81,82]. This mechanism of gene delivery by polymer-based vectors is illustrated in Figure 5 [83].

The polymers used in gene delivery have their own unique characteristics. Furthermore, they can be engineered to bring about desired characteristics in them and improve their efficiency of gene delivery. Targeting ligands can also be attached to these polymers to make them specified for a particular receptor found at the target site. They are also inserted with shielding reagents such as polyethylene glycol (PEG) that have the function of improving the time of vector circulation in the blood and allowing the safe contact of the polyplex with target cells. Many of these polymers are available on the market in a form ready to be used for gene delivery [84].

Some of the polymers used as cationic polymers for formation of polyplexes for gene delivery include polyethyleneimines (PEI), poly-l-lysine (PLL), and poly 2-*N*-dimethylaminoethyl methacrylate (PDMAEMA). These polymers exist in different structural forms such as branched, linear, star-shaped, hyperbranched, and dendritic structures [85]. Each of these structure exhibits its own characteristics which are employed in the preparation of cationic polymers for gene delivery [86]. However, the cytotoxic nature of these polymers limits their applications in gene therapy. Studies have reported that the cytotoxicity of these polymers is induced by their characteristics of size and surface charge. The large molecular weight of these polymers and inability to degrade their unnecessary charged groups and bonds makes them accumulate in normal body cells which causes the disruption of normal cell physiology, leading to cell cytotoxicity [87]. Furthermore, the complex structure of these polymers makes their clearance very difficult, which can further result in complexities. As a result of these aforementioned challenges, the use of these polymers as cationic polymers has reduced, and scientists are now focusing more on the use of biodegradable polymers to serve as cationic polymers that form polyplexes and deliver genetic material for gene therapy [84].

For more than 20 years, medical science has been making use of biodegradable polymers as carriers, vectors, and transmission agents for delivery of drugs and in many other treatment strategies such as gene therapy, regenerative medicine, biotechnology, genetic engineering, and tissue engineering. Among all these applications, biodegradable polymers are more relevant for gene delivery as they allow repeated administration and use, which is a significant aspect of gene therapy [88].

Biodegradable polymers can be natural or synthetic according to their origin. Both natural and synthetic biodegradable polymers have their own characteristics, advantages, and disadvantages which are explored and thoroughly studied before selecting a particular polymer for gene delivery. The main advantages of natural biodegradable polymers include their compatibility with biological environment, safe bioactivity levels, and ability to be proteolytic degraded by cell activation [89]. However, the precise extraction, purification, identification, and characterization of natural polymers acquired from natural sources is quite difficult, which can often result in variations in every batch of products made from them [90]. Such problems can later result in difficulty in gene therapy because of imperfect identification and determination of the composition of the polymers. This can have a very negative biomedical influence. Furthermore, the high levels of bioactivity of these polymers can result in triggering of a strong inflammatory or immune response which can be life threatening in some cases [91].

The main advantages of synthetic biodegradable polymers include their natural inert property, which makes them feasible for use in biological environment, and their controllable chemical structure, which allows structural modifications as per requirement of a specific therapy. This property also exhibits substantial uniformity in every batch produced by these polymers in contrast to natural polymers [92]. However, a drawback associated with these polymers is that they are biologically inert, thus hindering the preparation of these polymers for a specific biological therapy. Scientists and medical researchers are focusing on ways to incorporate different functional groups and chemical moieties in synthetic polymers to prepare them for targeted drug and gene delivery [93].

##### Protein-Based Vectors

Naturally biodegradable polymers are derived from two sources, i.e., proteins and polysaccharides. Proteins are made up of polypeptide molecules. They have a three-dimensional structure that is folded. Proteins make up a large number of biological tissues such as skin, hair, vascular tissues, and musculoskeletal tissues of humans, as well as bovine, equine, and porcine species. These proteins can also be obtained from sources such as fermentation from microbes and blood plasma [94]. These protein-based polymers act as amazing vectors for nonviral gene delivery owing to their remarkable biocompatibility and biodegradability.

The most common protein-based vectors used in gene delivery are gelatin and albumin. Gelatin polymers have great antigenicity properties, which make them very suitable for gene delivery. Kim and coworkers prepared gelatin-based nanoparticle vectors for the delivery of polymerized siRNA. These nanoparticles not only protected the siRNA genetic material from environmental degradation but also efficiently delivered them to melanoma cells bringing about sufficient gene expression in tumor-bearing mice models [95]. Chougule and coworkers formulated a gelatin-based nanocarrier formulation for delivery of a specific RNA called STAT6 siRNA to A459 cancer cells to inhibit the development and expression of STAT6 gene. They observed efficient gene silencing and destruction of the A459 cancer cells, which proved the efficiency of gelatin-based nanovectors for gene delivery [96].

Albumin has also been used in combination with some other cationic polymers for nonviral gene delivery. This is because albumin itself does not have a positive surface charge and, thus, needs cationic groups in order to impart the charged nature. Syga and her research group prepared a polyplex by combining plasmid DNA with PEI before insertion into albumin. The resulting nanovectors proved to be very efficient in transfecting the genetic material in the form of plasmid DNA to He La cells, and albumin was the major ingredient found to provide this efficient transfection ability [97].

##### Polysaccharide-Based Vectors

Polysaccharides refers to complex molecules made up of glycosidic linkage-bonded glucose units. These polymeric molecules have very efficient cell signaling properties, as well as capability of immune recognition. The presence of these characteristics has convinced scientists to explore these molecules for application in nonviral gene delivery. The presence of reactive functional groups on glucose units of these molecules makes them suitable for chemical modifications leading to formation of polysaccharide-derivatives which can be used for different gene delivery purposes. The most common biodegradable polysaccharide-based vectors are chitosan, β-cyclodextrin, dextran, and hyaluronic acid [98].

Chitosan and its derivatives are the most commonly used polysaccharides for gene delivery purposes because of the presence of randomly distributed β-1-4-linked d-glucosamine and *N*-acetyl-d-glucosamine groups that make it suitable for cationic polymer-based gene therapy [99]. However, the low pKa value of chitosan makes it much less soluble and hinders its interaction with genes. To combat this challenge, chitosan is mostly chemically modified to improve its efficiency of delivery and transfecting genetic material. Kean and his research group developed trimethyl chitosan-based nanovectors by modification of chitosan backbone. They saw efficient delivery of plasmid DNA to cancer cells using this method [100].

Other natural polysaccharide-based biodegradable polymers used as nonviral vectors include dextran, hyaluronic acid (HA), and β-cyclodextrin. These polymers lack cationic groups of their own, as a result of which they require to be combined with other cationic polymers [101].

##### Polyesters

Biodegradable synthetic polymers are classified on the basis of the presence of chemical bonds in their backbone that are labile to hydrolysis. These chemical bonds, esters, amides, urethanes, anhydrides, and carbonates result in the nomenclature of the corresponding polymers, i.e., polyesters, polyamides, polyurethanes, polyanhydrides, and polycarbonates [102].

Polyesters are usually aliphatic in nature and have largely been employed in medical science in sutures, genetic engineering, carriers for drug delivery, and gene therapy. A large number of polyester polymers containing different types of monomer units are being explored for potential applications in gene delivery. The most commonly used polymers in this respect are PPE, PHP, PVL, PAGA, and PBAE [91].

The backbone of PPE polymers is compatible and recognizable with biological enzymes and is also analogous to nucleic acids. This property makes them very suitable for gene delivery. Although these polymers possess very efficient biodegradable properties, their complex molecular structures hinder gene delivery preparation. For this purpose, scientists have developed various techniques to modify these polymers and bring about enhanced transfection efficiency and reduced toxicity [103]. An example of this is the formation of a copolymer micellar system that shows much greater transgene delivery than simple polyester polymers. Many studies employed the use of micelle formation by combining three different polyester polymers to deliver siRNA to target cells for treatment of hypoxic tumors and other types of cancer. They showed that use of micelles of these polymers as vectors not only efficiently encapsulated and protected the gene cargo but was also capable of effectively delivering the transgene to target site and bringing about successful gene expression [104,105,106].

##### Polycarbonates

The increased biocompatibility, decreased cytotoxicity, and controllable mechanical properties make polycarbonates efficient gene delivery vectors [107]. An example of chemical modification of polycarbonates for gene delivery was demonstrated by Frere and coworkers. They produced a series of new guanidinium- and morpholino-functionalized polycarbonate-based vectors that were biocompatible and biodegradable. These vectors were aimed at delivering siRNA to HeLa cells for the treatment of cervical cancer. They proved that the chemical modification provided the polycarbonate vectors with a greater degree of transfection and gene delivery capability [108].

The chemical modifications and combination with copolymers of polycarbonates not only enhanced their biodegradability but also their gene transfection ability, while also reducing the side-effects of immune reactions and cytotoxicity due to these polymers, making them efficient agents for nonviral gene delivery [109].

##### Polyurethanes

The specific properties of polyurethanes such as elasticity, biocompatibility, and flexibility make them highly suitable for applications in gene delivery and tissue engineering. Similar to other synthetic biodegradable polymers, polyurethanes are also suitable for gene delivery applications by combining them with other cationic polymers. An example of this was demonstrated by Yang and coworkers. They prepared a complex of polyurethane with PEI to act as vector for delivery of miRNA for the treatment of brain tumors. This complex not only delivered the genetic material very efficiently but was also capable of inhibiting proliferation of glioblastomas, in addition to being very effective in gene therapy of lung cancer [110].

In Table 3, advantages and disadvantages of polymer-based nonviral vectors of gene delivery were described.

#### 3.2.2. Lipid-Based Nanovectors

Research on gene delivery until now has come up with three main lipid-based systems for gene delivery. These include liposomes which are of further different types, microvesicular systems, and high-density lipoprotein-mimicking systems. All these systems have their own unique characteristics, and their ability to carry genetic material for gene delivery depends on their pharmacokinetic properties, biodistribution, properties of drug release, and release kinetics.

Recently, the potential of lipid-based nanosystems was studied for application in the development of SARS-CoV-2 vaccines. Lipid-based nanoparticles were of utmost interest in this regard. Even the first COVID-19 vaccine to enter the clinical trial phase was an mRNA-based vaccine that was delivered via the use of lipid nanoparticle vectors [111]. Similarly, Moderna’s COVID-19 vaccine is also based on lipid nanoparticles. In addition to this delivery platform, other lipid-based nanoparticulate systems considered for use in vaccine development of SARS-CoV-2 include cationic nanoemulsions and liposomes [112].

**Figure 6:**
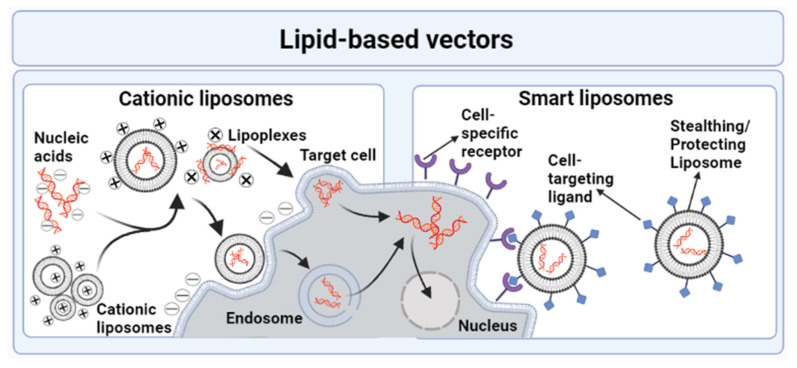
Gene delivery by lipid-based nonviral vectors.

The mechanism of gene delivery by lipid-based nanovectors is given in Figure 6 [83].

##### Liposomes

Liposomes refer to specialized particles of macro or nano size that contain one or more bilayers of lipids surrounding an aqueous core. The process of liposomal formation is based on self-assembly, whereby an ethanolic solution containing the lipids is combined with a nucleic acid-containing aqueous solution. The use of liposomes as vehicles for drug and gene delivery has been going on since the 1970s. The efficiency of liposomes as gene-carrying vectors depends on their physical and chemical properties such as size, composition, efficiency of loading, net charge, and stability [113].

Liposomes are of many different types, but the two main types relevant for gene delivery are cationic liposomes and smart/trojan horse liposomes, which are further explained below.

- Cationic Liposomes

This type of liposome possesses an overall positive charge, which makes it capable of combining with negatively charged genetic materials and nucleic acid via the use of electrostatic interactions. As a result of this interaction, the particles of nucleic acids that are larger in size are entrapped inside the lipoplex structure. The easy modification of cationic liposomes to enter the in vitro environment with a net positive surface charge makes them capable of combining with the negatively charged molecules of the cellular membrane, thus leading to cellular internalization and gene delivery. The fusogenic properties of liposomes enables the nucleic acid-carrying lipoplex to escape endosomal degradation and effectively delivery the gene into the cell cytosol or nucleus [114].

Cationic liposomes were first used in 1987 as vectors for gene delivery by Felgner and coworkers [115]. Since then, several researchers have explored different cationic lipids for the purpose of gene therapy and gene delivery. The various structural components of cationic lipids play an important individual role in enhancing the transfection capability of genetic material. If the cationic head groups of liposomes are made up of more than one amine, imidazole, or guanidine group, then they are capable of rendering a greater cationic nature to the overall lipoplex entity. Such multivalent cationic liposomes have greater ability to protect the genetic material from lysosomal degradation. However, too much positive charge can lead to very strong bonding between the nucleic acids and liposomes, which can cause a problem in dissociation of the genetic material from liposomes and their consequent release in the cytoplasm. Furthermore, multivalent liposomes are vulnerable to micelle formation and can also pose a danger to the stability of the liposome–nucleic acid complex, which can also lead to cytotoxicity. Such lipid-containing lipoplexes are also more difficult to metabolize, which makes their clearance after gene delivery a tedious task [116].

Some of the most significant benefits of using cationic liposomes as compared to the very complex trojan horse liposomes include their amazing efficiency of delivering nucleic acids in an in vitro environment, absolutely no limit to the size of genetic material that can be delivered through cationic liposomes, easy and simple manufacturing, and cost-effectiveness. Many liposomes are commercially accessible in the pharmaceutical market ready to be used for gene delivery [117].

The capability of cationic lipoplexes to aggregate and form micelles, as well as their unstable nature, tendency to combine with blood components, and poor distribution inside the body, makes then unsuitable for gene delivery via an intravenous route. These drawbacks not only reduce their transfection efficiency but also modify the charge of lipoplexes, which can cause them to accumulate in various organs, leading to organ toxicity and potential chronic harmful effects [118].

- Smart Liposomes

The capability to circulate for longer periods of time in the bloodstream and target specific cells makes any liposomal system ideal for gene delivery. These properties render the system capable of reaching the target site in the administered therapeutic dose and reducing the side-effects caused by the interaction of nucleic acids with healthy cells. These properties are generally exhibited by smart or trojan horse liposomes, also called stealth liposomes. They show long permanence in the blood stream and are also very much able to evade the immune system and trigger unwanted immune responses. Liposomes can achieve an increased time of blood circulation by introducing hydrophilic polymers such as polyethylene glycol to their surface. This makes the liposomal complex inert toward blood components and other blood circulating entities, thus preventing their opsonization and elimination by the reticuloendothelial system [119].

A unique ability of smart liposomes which makes them different is their capability delivery the genes or genetic material to a specific cell group or population which otherwise needs to be achieved using targeting ligands on the nanovector surface. An example of such ligands is the transferrin protein, which is specifically used to target cancer cells where the transferrin receptor is overexpressed [120]. Smart liposomes are also capable of responding to different stimuli and modifying their gene delivery efficiency accordingly. Stimuli such as pH [121], light [122], magnetic field [123], temperature [124], redox reactions [125], and ultrasound [126] have the tendency to cause liposomal destabilization, leading to the release of gene cargo from these carriers. This stimulus-responsive property can be utilized to develop controlled release systems that will deliver their cargo only upon response to a particular stimulus at the target site [127]. In Table 4, the advantages and disadvantages of lipid-based non-viral vectors of gene delivery are presented.

#### 3.2.3. Inorganic Materials

**Figure 7:**
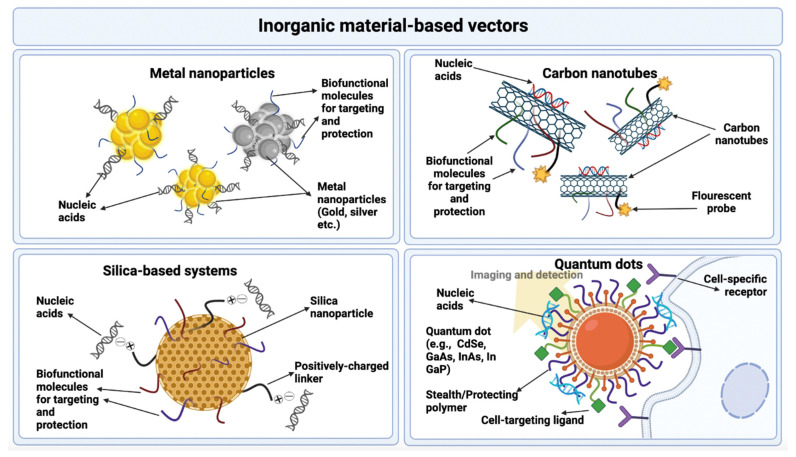
Gene delivery by inorganic material-based vectors.

A large number of inorganic nanoparticles are synthesized using various inorganic materials. The physical and chemical properties exhibited by these nanoparticles utterly depend upon their composition. Some of the inorganic materials most commonly used to make inorganic nanoparticles as drug and gene carriers are iron oxide and its compounds, metallic compounds, quantum dots, carbon nanotubes, and silica-based compounds that come under graphene-based systems. These systems pose many advantages which make them feasible as vectors for gene delivery. These include remarkable biocompatibility, negligible cytotoxicity, easy handling and convenient functionalization [128]. The further advantages and disadvantages of the use of inorganic materials in gene delivery are given in Table 5, and these materials as gene delivery vectors are presented in Figure 7 [83].

##### Metal Nanoparticles

Metal nanoparticles have recently emerged as excellent vectors for gene delivery. The most commonly used metals used in the preparation of these nanoparticles are gold and silver. These nanoparticles pose specific advantages which make them distinguished and better than other nonviral gene delivery systems [129]. They exhibit specific plasmon resonance on the surface that enables them to be more sensitive to the environment. They have an easily modifiable composition and surface functionality, allowing them to be used for specific targeting and other such applications [130]. The synthesis process of these substances is simple. They show amazing biocompatibility and reduced cytotoxicity. After administration, these particles can be easily tracked via the use of various methods such as fluorescence resonance energy transfer (FRET) [131].

##### Quantum Dots

Quantum dots are crystals made from semiconductor materials. They have a size ranging from 1 to 20 nm. They consist of a large number of substances ranging from hundreds to thousands in number that are arranged in the form of groups or clusters. These compounds can be binary such as SiC and GaAs or ternary such as InGaAs and InGaN. The main usage of quantum dots is as fluorescent probes, and they present many advantages as compared to other common probes. These include their increased brightness, prolonged fluorescence time, and excessive photostability [132].

Quantum dots are used for gene delivery purposes as the core of the gene carrying vector. Nucleic acids and other targeting ligands are attached to the surface of this core. This leads to a combination of the therapeutic ability of the genes with the fluorescent ability of the quantum dots, which makes this system theranostic. Using this method, the administered nucleic acids can be tracked with the help of these quantum dots after their entrance into the body [133].

##### Carbon Nanotubes

Carbon nanotubes consist of sheets of graphene arranged in a cylindrical, tube-like shape. Their structure can be based on single-walled tubes with a diameter of 0.4–3 nm or multiwalled tubes with a diameter of 4–30 nm. These systems exhibit great benefits including a great capacity of carrying drugs, chemical inertness, and tendency to provide controlled drug release. They also allow the conjugation and attachment of other biomolecules, targeting ligands, and probes that provide fluorescence. In this way, their ability to provide drug and gene delivery can be improved, and the delivery process can be monitored by use of probes. They also form very stable complexes upon conjugation with nucleic acids, thus ensuring their safe delivery to target cells. A drawback of these substances is that they are very incompatible and insoluble in aqueous environments which makes the application for biological use a bit complex [134].

##### Silica-Based Systems

The use of compounds based on silicon and its derivatives such as silicon dioxide has been prominent for gene and nucleic acid delivery for many years now. Nanoparticles made from this system can be modified in terms of their surface charge, size, shape, and other properties to make them suitable for attachment with several targeting ligands and other biomolecules for targeted drug and gene delivery. Thus, properties such as persistent blood circulation, efficient transfection, low toxicity, improved capacity of drug loading, and enhanced cellular internalization can be achieved in these nanoparticles by modification. Furthermore, these substances are easily available commercially, are cost-effective, and do not require complex manufacturing procedures. The biggest advantage of these systems is that they allow the storage of a large amount of DNA and genetic materials, and they are compatible with both hydrophobic and hydrophilic substances, which is quite unachievable in other carrier systems [135].

Cationic substances can be added to the surface of these silica-based systems to make them capable of conjugating with the negatively charged DNA to deliver them to target sites. They can also be modified to bring about stimulus-responsive properties, allowing them to be used for noninvasive gene delivery methods. The two main advantages of these systems observed by research are their ability to combine with the membranes of blood cells, causing hemolysis [136] and metabolic changes with can result in melanoma [137].

## 4. Conclusions

Over the last 10–20 years, gene therapy has been gaining significant attention in the world of medical science. With its capability to cure numerous untreatable diseases such as AIDS, Parkinson’s disease, lysosomal storage disorders, and other genetic and acquired diseases, gene delivery has revolutionized modern therapeutics. Viral and nonviral vectors each have their own advantages and disadvantages. The choice of a vector is based upon the nature of the gene to be delivered, the physical and chemical characteristics of the vector, and the condition to be treated. With continuous research on vectors used in gene delivery, new and more efficient viruses and nonviral materials are emerging as potential carriers having greater gene transfection ability with fewer side-effects. The employment of innovative strategies in finding and developing multifunctional vectors of high efficiency for combating barriers in gene delivery has been very beneficial to progress in this field. However, still more research is required with respect to clinical trials for testing new and innovative vectors that can provide greater gene transfection ability and a persistent, sustainable gene expression with the lowest potential of cytotoxicity. Furthermore, additional knowledge is vital for a better understanding of transfection mechanisms, so that more possibilities of vector modification can be explored to optimize the gene delivery process. Moreover, new ways of combining various types of vectors need to be researched so that their advantages can be synergized for achieving optimal gene transfection results. Genetic engineers and gene therapists are entitled to finding rational ways of combating gene delivery barriers, preparing vectors for optimal gene delivery, and uncovering ingenious vectors with a greater potential of efficient gene delivery.

## Figures and Tables

**Table 1:** Advantages and disadvantages of viral vectors.

Advantages	Disadvantages
Provide greater gene transfer efficiency in both in vivo and in vitro environments	Can trigger severe immune responses and inflammatory reactions
Persist for longer periods of time in most cases	Their cloning capacity is very limited
Can target a large number of cells	Produced by complex production methods
A large variety of viruses are available to choose from	Low capability of tropism to some specific target cells
Innate ability of tropism toward infection	Can cause mutagenesis by inserting their exogenous DNA into the host genome
Capable of evading endosomes by various mechanisms learned by evolution of viruses	Research is needed to further understand the mechanisms of molecular infection by viruses

**Table 2:** Advantages and disadvantages of physical methods of nonviral gene delivery.

Techniques	Advantages	Disadvantages
Microinjection	Allows delivery of large amount of genetic material, convenient, simple, cost-effective, less toxic, and reproducible	Special handling technique is required and cannot be used for large number of cells transfection
Needle injection	Simple to perform and requires small amount of DNA	Therapeutic efficacy is quite low and is difficult to conduct
Jet gun	Noninvasive, safe, and easily controllable	Causes local tissue damage and efficiency is low
Gene gun	Nontoxic, highly effective, and allows gene delivery to cells that are difficult to transfect	Limited to superficial cells and cannot be used for gene delivery to cells where deep penetration is required
Electroporation	Fast, effective, reproducible, and allows delivery of large quantities of DNA	Requires surgery, risk of DNA damage due to exposure to high voltage, and highly localized
Nucleofection	Fast and efficient in cases where cell membranes are difficult to permeate	Very limited application for in vivo gene delivery and can be highly toxic
Sonoporation	Noninvasive, capable of reaching deep tissues and organs, can be used for specific local targets, and capable of crossing blood–brain barrier	Efficiency is relatively low and target cells can be damaged
Hydrodynamic gene transfer	Simple and very efficient in deliver of gene to liver cells	Injection volume required is very large and clinically not feasible
Magnetoporation	Noninvasive and capable of reaching cells that are deep and demand complex transfection	Special equipment is required, preparation of magnetic vectors is complex, and magnetic reagent can cause toxicity after removal of magnetic field
Mechanical Massage	Simple, noninvasive, and easy to apply	Efficiency is low and application is not yet available for humans

**Table 3:** Advantages and disadvantages of polymer-based nonviral vectors of gene delivery.

Polymer-Based Vector	Advantages	Disadvantages
Protein-based vectors	Highly biocompatible, biodegradable, and non-toxic	Have low mechanical strength and are vulnerable to rapid degradation by biological components
Polysaccharide-based vectors	Highly biocompatible, biodegradable, hydrophilic, nontoxic, and easily modifiable with ligands and functional groups	Lack cationic groups of their own and need to be modified to make them interact with genetic materials
Polyesters	Have a compatible and biologically recognizable backbone that is analogous to nucleic acids	Complex molecular structure that is difficult to study and modify
Polycarbonates	Nontoxic, highly biocompatible, and controllable mechanical properties	Need to be modified with ligands to avoid unwanted immune reactions
Polyurethanes	Highly elastic, flexible, and biocompatible	Need strict control of molecular weight to form complex with DNA

**Table 4:** Advantages and disadvantages of lipid-based nonviral vectors of gene delivery.

Lipid-Based Vector	Advantages	Disadvantages
Cationic liposomes	Fusogenic, compatible with DNA, and can carry large amount of genetic material	Are unstable and can result in aggregation, micelle formation, poor distribution, and toxicity via intravenous route
Smart liposomes	Stealth properties and ability to circulate in bloodstream for longer periods	Surface needs to be coated with polymers or protective substances to prevent interaction with or degradation by blood components

**Table 5:** Advantages and disadvantages of inorganic materials used in gene delivery.

Inorganic Material	Advantages	Disadvantages
Metal nanoparticles	Allow molecular tracking after administration, can be modified to achieve targeting, biocompatible, and easy manufacturing	Special equipment is required for preparation
Quantum dots	Capable of conjugating with many ligands and biomolecules for specialized targeting, and very sensitive to tracking probes	Production mechanism is complex and requires sensitive handling
Carbon nanotubes	Nanosized, amazing capacity to load drugs, remarkable efficiency, and chemically inert	Poor aqueous solubility, complex manufacturing, and expensive
Silica-based systems	Allow vast chemical modification, low cytotoxicity, good storage capacity, stable, and amazing capacity to load drugs	Can cause disruption in metabolic process, toxicity, and hemolysis

## Data Availability

Not applicable.
